# MiRmat: Mature microRNA Sequence Prediction

**DOI:** 10.1371/journal.pone.0051673

**Published:** 2012-12-27

**Authors:** Chenfeng He, Ying-Xin Li, Guangxin Zhang, Zuguang Gu, Rong Yang, Jie Li, Zhi John Lu, Zhi-Hua Zhou, Chenyu Zhang, Jin Wang

**Affiliations:** 1 The State Key Laboratory of Pharmaceutical Biotechnology and Jiangsu Engineering Research Center for MicroRNA Biology and Biotechnology, School of Life Science, Nanjing University, Nanjing, China; 2 National Key Laboratory for Novel Software Technology, Nanjing University, Nanjing, China; 3 Institute of Machine Vision and Machine Intelligence, Beijing Jingwei Textile Machinery New Technology Co., Ltd., Beijing, China; 4 Biophysics Institute, School of Physics, Nanjing University, Nanjing, China; 5 Ministry of Education Key Lab of Bioinformatics & System Biology, School of Life Science, Tsinghua University, Beijing, China; University of Alberta, Canada

## Abstract

**Background:**

MicroRNAs are known to be generated from primary transcripts mainly through the sequential cleavages by two enzymes, Drosha and Dicer. The sequence of a mature microRNA, especially the ‘seeding sequence’, largely determines its binding ability and specificity to target mRNAs. Therefore, methods that predict mature microRNA sequences with high accuracy will benefit the identification and characterization of novel microRNAs and their targets, and contribute to inferring the post-transcriptional regulation network at a genome scale.

**Methodology/Principal Findings:**

We have developed a method, MiRmat, to predict the mature microRNA sequence. MiRmat is essentially composed of two parts: the prediction of Drosha processing site and the identification of Dicer processing site. Based on the analysis of microRNAs from 12 species, we found that the patterns of free energy profiles are conserved among vertebrate microRNA hairpins. Therefore, we introduced in our method the free energy distribution pattern of the downstream part of pri-microRNA secondary structure and Random Forest algorithm to predict the mature microRNA sequence. Based on the evaluation on an independent test dataset from 10 vertebrates, MiRmat was shown to identify 77.8% of the Drosha processing sites and 92.8% of the Dicer sites within a deviation of 2 nt. In a more stringent evaluation by excluding the microRNAs sharing the same family between the training set and test set, MiRmat kept a rather well performance of 71.9% and 87.2% of the identification rate on the Drosha and Dicer site respectively, which represents the ability to deal with the novel microRNA family. MiRmat outperforms other state-of-the-art methods and has a high degree of efficacy for the prediction of mature microRNA sequences of vertebrates.

**Conclusion:**

MiRmat was developed for identifying microRNA mature sequence(s) by introducing the free energy distribution of RNA stem-loop structure and the Random Forest algorithm. We prove that MiRmat has better performance than the existing tools and is applicable among vertebrates. MiRmat is freely available at http://mcube.nju.edu.cn/jwang/lab/soft/MiRmat/.

## Introduction

MicroRNA (miRNA) is a class of small, non-coding RNAs that are crucial for the development of many species and are frequently involved in a variety of genetic diseases, including cancer [1], [2], [3], [4]. Generally, microRNAs function through the down-regulation of gene expression via a targeting mechanism on mRNA [5]. Mammals are believed to share a common mechanism of microRNA biogenesis [6]. First, the primary microRNAs (named pri-microRNAs) are transcribed from genome and processed into precursor microRNAs (pre-microRNAs) in the nucleus by a Microprocessor complex which is composed of Drosha (an RNase III enzyme) [7] and its cofactor DGCR8 (also known as Pasha) [8]. Pre-microRNAs have a typical hairpin structure of ∼60–70 nt, which is characterized by an overhang of ∼2 nt at the 3′ end [9]. The pre-microRNA is then transported to the cytoplasm by a pre-microRNA-specific export carrier, Exportin 5, which is accompanied by a Ran-GTP cofactor [10], [11], [12]. The pre-microRNA is subsequently cleaved by Dicer (another RNase III enzyme) to yield a microRNA duplex with 3′-overhang of ∼2 nt [13], [14], [15]. Usually, one strand of the duplex remains as the mature microRNA and is incorporated into a RNA-induced silencing complex (RISC). The RISC will then target mRNAs based on partial sequence complementarity [16]. Obviously, the sequence of mature microRNA plays the key role in the recognition of targets [17], [18], [19]. The processing steps mentioned above are taken by most microRNAs, although some studies showed that a small subgroup of microRNAs that are located in short introns can bypass the Drosha step via another pathway named miRtron [20].

Experimental approaches such as sequencing and cloning are widely used for identifying numerous microRNAs in their mature state [21], [22], [23]. However, these types of methods are inevitably biased towards those microRNAs that are abundantly expressed. The recent deep sequencing technique has been proven to be efficient in discovering novel microRNAs, yet, to find out the real microRNAs from the huge number of ‘reads’ that are produced from Solexa or other deep sequencing machines becomes another challenge [24], [25].

Most of the computational methods developed so far aim to identify potential microRNA genes from the genomes of different species [9], [26], [27], [28], [29] by identifying the stem-loop structures that contain pre-microRNAs, instead of predicting the precise mature sequence of microRNAs, although some of them do include the study of mature sequence as an inline procedure [9], [26], [30]. For example, sequence alignments were involved in miRseeker [26] to determine the regions that encompass the mature microRNA. In proMIR [9], a hidden Markov model (HMM) was introduced to find mature microRNA region. Then, Microprocessor SVM was carefully designed to predict the Drosha processing site that determines one end of the mature microRNA in human genome by using over 600 features for the SVM classifier [13]. Recently, MatureBayes was reported which specifically identifies the mature microRNA [31]. This tool uses a Naïve Bayes Classifier to predict the start position of mature microRNA on the precursor and then determines the mature sequence according to the length of 22 nt.

Here, we present a new tool, MiRmat, for identifying the sequence of mature microRNA from the stem-loop structure that exists in the pri-microRNA. The method is based on the principle of free energy in molecule interaction and the process of mature microRNA biogenesis. Firstly, the free energy distribution pattern of the stem-loop structure derived from the pri-microRNA is introduced for the prediction of Drosha processing sites, i.e., the prediction of precise pre-microRNA. Then, the structural features of pre-microRNA are applied for the prediction of Dicer processing site, so that the mature microRNA sequence is produced.

A pri-microRNA may fold to the secondary structure that contains one stem-loop (or more stem-loops) which is typically a stem of ∼33 bp with a loop at one end and flanking single strands at the other end. Normally, it is hard to know the precise length of a pri-microRNA [32], [33], [34]. For convenience, such kind of stem-loop structures that contains the corresponding pre-microRNA is commonly called the microRNA hairpin or microRNA stem-loop. In this paper, we regard the side with the terminal loop as upstream while the opposite direction is termed as downstream. According to the previous studies, Drosha binds to pri-microRNAs through dsRBD (Double-stranded RNA-binding domain) accompanied by a partner molecule DGCR8, which helps to anchor the Drosha Microprocessor complex to the correct position on the pri-microRNA structure [35]. Han, J. et al. (2006) calculated the thermodynamic stability profiles of pri-microRNA secondary structures and suggested that a distance of about 11 bp downstream from the stem-ssRNA junction is likely to be Drosha processing site, while the upstream region of the structure near the loop is of little significance [36]. Obviously, a precise determination of Drosha processing sites could not simply rely on this value, as the actual profiles calculated from the sequences and structures vary significantly among microRNA hairpins. In MiRmat, we build a new model to analyze the patterns of free energy profile of microRNA hairpins and apply the Random Forest (RF) algorithm to identify the Drosha processing site. Based on an analysis on microRNAs of 12 species we found that the free energy pattern derived from our model may be conserved among vertebrates.

While the Drosha processing site determines one end of the mature microRNA sequence, the other end of the mature microRNA sequence is determined by the Dicer processing site. Dicer is an enzyme of multiple domains including an N-terminal DexH-box RNA helicase-like domain, a PAZ domain, two RNase III domains (RNase IIIa and RNase IIIb), a dsRBD and a domain of unknown function (DUF283) [37]. According to a previous model, Dicer serves as a molecular ruler that measures and cleaves several nucleotides from the end of a dsRNA [38]. The length of the products is determined by the distance between the RNase III domains and the PAZ domain, which is about 65 Å in *Giardia intestinalis* and matches the length spanned by 25 bp of RNA [38]. Yet, this length is easily influenced as the enzyme frequently induces a conformational change of the double-stranded RNA for an efficient and precise cleavage. Through the statistics pertaining to pre-microRNA secondary structure and the length of mature microRNA sequence, we found the possible relations between the two parameters. Therefore, a set of structural features were selected for applying Random Forest to identify the Dicer processing sites on pre-microRNAs.

By sequentially applying the free energy distribution patterns of the secondary structures of microRNA hairpins and the structural features representing the length of mature microRNA sequence, MiRmat has been able to identify 31.9% of the Drosha sites for vertebrate microRNAs at exactly the annotated positions provided by miRBase and the Dicer processing site with a rate of 45%. If a deviation of 2 nt from the true site is allowed, the rate of identified sites for Drosha approaches 80% while the prediction rate for Dicer site exceeds 90%. Based on the tests of the same datasets, MiRmat shows better performance than the other existing methods.

## Results

We proposed a new method for microRNA mature sequence prediction and developed the new tool, MiRmat. Generally, MiRmat is combined by two sequential steps. The first is for Drosha processing site prediction, which uses the energy distribution pattern as the feature and Random Forest (RF) algorithm as classifier. The feature of energy distribution pattern was derived from the conservation analysis of the free energy distribution along the stem-loop structure of pri-microRNAs. The second step is for Dicer processing site prediction, which uses RF algorithm to recognize the structural features. By predicting Drosha and Dicer processing sites, MiRmat can finally obtain the microRNA mature sequences. The details in the two steps are described in the section ‘Materials and Methods’.

### The Energy Distribution Pattern of microRNA Hairpins

**Figure 1:**
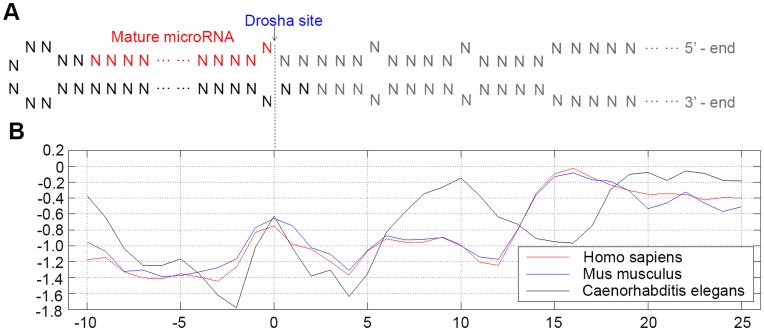
Energy distribution patterns of the microRNA stem-loop structure of 3 species. (A). Diagram of microRNA hairpin structure; (B). Energy distribution pattern of 3 species: *H. sapiens* (red), *M. musculu*s (blue) and *C. elegans* (black).

Fig. 1 plots the free energy distributions along the stem of the microRNA hairpin structure of 3 organisms (based on miRBase 9.2). The horizontal axis represents the distance of a stack face (the plane formed by base pair stacking) away from the Drosha cutting site. Zero indicates the stack face at the Drosha cutting site. Minus refers to upstream (i.e., the direction to the loop of hairpin). The red line and blue line show the free energy distribution along the microRNA stem-loop structure for *H. sapiens* (hsa) and *M. musculus* (mmu) respectively, while the black line represents the energy distribution pattern for the invertebrates, *C. elegans* (cel). An analysis of the energy distribution pattern of a total of 10 vertebrates and 2 invertebrate is shown in (Figure S1). It is clear that all vertebrates have, to some extent, similar free energy distribution pattern (e.g., a low free energy distribution in the region 10 to 13 nt and an energy peak in the region 15–20 nt) with fluctuations though, whereas the invertebrates do not have this characteristic pattern. The average correlation coefficient between the patterns of *H. sapiens* and the other 9 vertebrates is 0.9395 in the region 0 to 19 nt, while the correlation coefficient for that of *H. sapiens* and *C. elegans* is 0.2435. The method proposed here involves Random Forest algorithm to identify the pattern that determines the mature microRNAs from the fluctuating energy distribution data.

### Performance of MiRmat on the Prediction of Drosha Processing Site

#### Performance evaluation

The prediction power of MiRmat in identifying Drosha processing sites was evaluated on the test set retrieved from miRBase as described in Materials and Methods. To obtain reliable testing result, non-redundant samples were prepared by excluding all the hairpin sequences with a similarity of over 90%.

**Figure 2:**
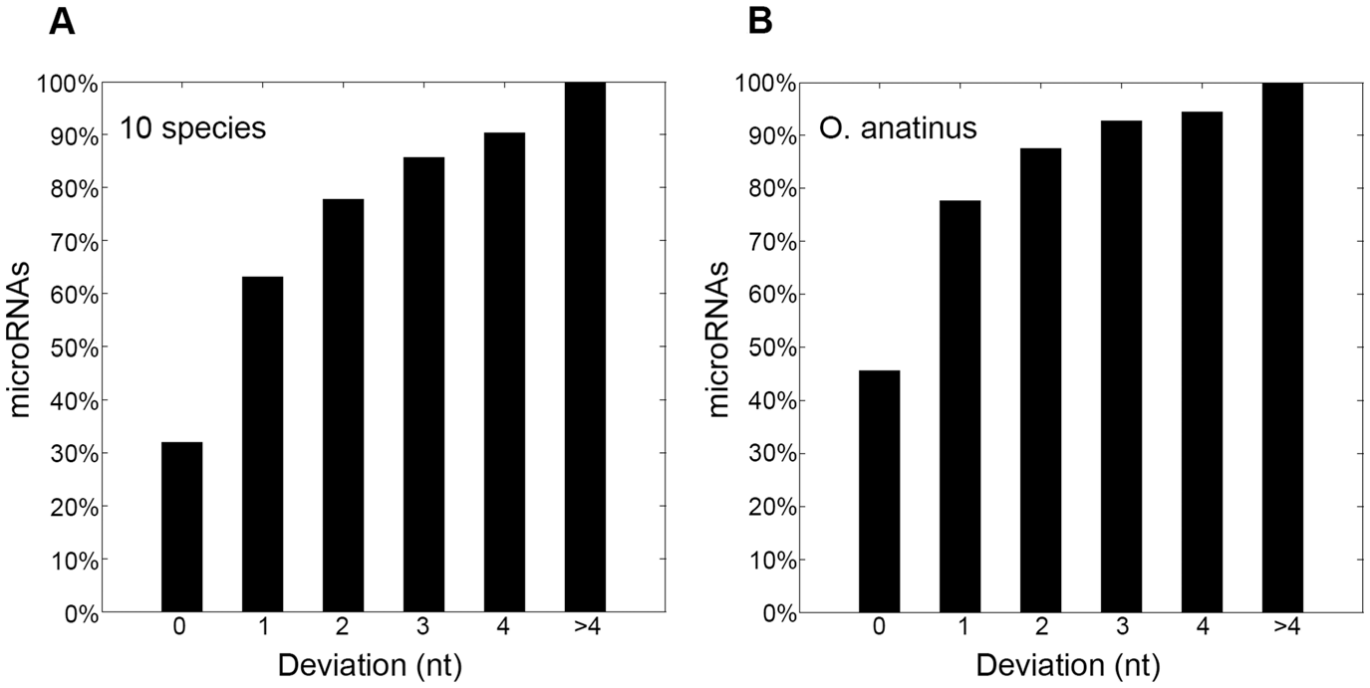
Rate of correct predictions of Drosha processing site. ‘Deviation (nt)’ means the distance between the predicted Drosha processing site and the true site. (A) Performance on microRNAs of 10 vertebrates in the test dataset (*M. domestica, H. sapiens, M. mulata, M. musculus, P. troglodytes, R. norvegicus, G. gallus, X. tropicalis, O. anatinus, C. familiaris*); (B) Performance on *O. anatinus* microRNA data set.

Fig. 2A shows the performance of MiRmat on Drosha processing site prediction. The Drosha sites of 31.9% of microRNAs in the test set were exactly predicted according to the annotations in miRBase. If a deviation of 2 nt from the true site is allowed, the rate of successful prediction reaches 77.8% (Fig. 2A). It should be noted that, like the previous study [13], MiRmat is also limited to deal with microRNA samples with the regular structure that the lengths of the pre-microRNAs are within 50–80 nt and the mature sequences locate in the stems.

To test the performance of MiRmat on novel microRNA families, a more stringent evaluation was made by excluding all the samples in the test set that belong to the same family as that in the training set. The microRNA family data was downloaded from miRBase at ftp://mirbase.org/pub/mirbase/CURRENT/miFam.dat.gz. According to the definition of microRNA family, these are the homologs from the same ancestor in principle. In this case, the prediction rate still keeps at 71.9% (Figure S2A), indicating that the model involved in MiRmat generalizes well over both different vertebrate species and different microRNA families.

#### Performance of MiRmat on an independent validation set

The microRNAs of platypus, *O. anatinus* (oan), were collected after miRBase version 9.2 was released. Therefore, we used these data as an additional independent validation set to evaluate the performance of MiRmat. As shown in Fig. 2B, MiRmat predicted the Drosha processing sites of *O. anatinus* microRNAs with a fairly high accuracy; 87.5% of the processing sites were predicted within 2 nt of the true sites. This means that the free energy pattern derived from other vertebrate species is also applicable to *O. anatinus* microRNAs. Because platypus represents a fascinating combination of reptilian and mammalian traits [39], this result further supports that the energy distribution pattern downstream the Drosha processing site may be conserved among vertebrates. When all the samples homologous to that in the training set were removed from the test set, MiRmat identified 82.4% of the Drosha sites that are from the novel families. This result was presented in Figure S2B.

#### Tests on random sequences

We tested the specificity of MiRmat by applying the tool to randomly collected hairpins and comparing the average scores resulted from the Random Forest algorithm with the scores for true microRNAs.

We generated the random hairpins from the 3′UTR sequences as the negative control as it was reported that microRNAs are extremely rare in 3′UTRs [29]. The 3′UTR sequences were downloaded from UTRdb [40]. The secondary structures were predicted by RNAfold [41]. The criteria for selecting the test hairpins include: 1) no more than 18 base pairings on the stem; 2) a maximum free energy of −15 kcal/mol of the secondary structure [42]. These criteria ensure that these hairpins are similar to the true microRNA hairpins in terms of general structural characteristics. Two sets of hairpins were constructed by randomly picking one qualified hairpin from each 3′UTR sequence. The first set, RHD-1, contains 1815 hairpins from mammalian 3′UTR sequences (except for *H. sapiens* 3′UTRs), and the second dataset (RHD-2) contains 470 qualified hairpins from human chromosome 1.

As shown in Table 1, MiRmat has a significantly higher Random Forest score (p<<0.001) on the microRNA hairpins than on the non-microRNA samples, which means the energy distribution pattern of microRNA hairpins is significantly different from those of non-microRNA hairpins.

**Table 1:** Average RF scores resulted from different test sets.

Datasets	Test set^a^	RHD-1^b^	RHD-2^c^
Average RF score	0.3110	0.2416	0.2403

a vertebrate microRNA hairpins;

b mammalian hairpins;

c human hairpins.

### Performance of MiRmat on the Prediction of Dicer Processing Site

The performance of MiRmat on predicting Dicer processing site was tested on the pre-microRNAs that are annotated in the miRBase. Limited by our prediction model, the samples with irregular structures, i.e., the annotated Dicer site is on the terminal loop of pre-microRNA structure or the internal loops is too long (>6 nts), were removed from the training and test sets. The test was also limited to the microRNAs with the mature sequence of 16–25 nt.

**Figure 3:**
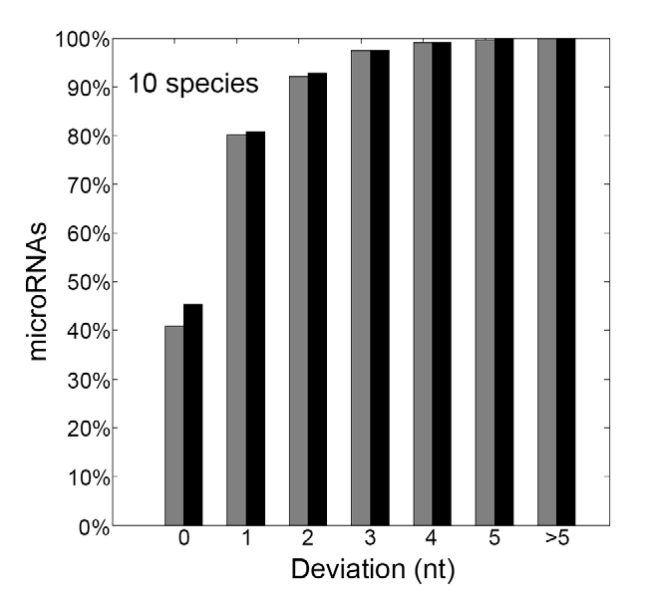
Rate of correct prediction of Dicer processing site. The black column represents the accuracy of MiRmat by using all the 42 features; the gray column shows the performance of MiRmat by using the length feature.

Fig. 3 plots the performance of MiRmat on Dicer processing site prediction. The black columns show the accuracy of MiRmat by using all the 42 features: of the 1269 microRNAs in the test set, MiRmat could correctly predict 576 (45.4%) processing sites; 1178 (92.8%) true processing sites were within 2 nt of the predicted processing sites. The gray columns show the performance of MiRmat by only recruiting the length of mature sequence (last feature in Table 2) as the feature. As shown in Fig. 3, the length of mature microRNA sequence determines the approximate position of Dicer processing site, while the secondary structure of pre-microRNA influences the precise position of the cutting site. Among the test dataset, ∼60 more Dicer sites were precisely predicted by taking into account the structural features.

**Table 2:** Categories of features for Dicer site prediction.

Features	Number of Feature
Size of internal loops in candidate mature sequence region^a^	36 (6 x 6)
Size and number of bulges in candidate mature sequence region^b^	4
Position of first paired nucleotides of pre-microRNA structure^c^	1
Length of candidate mature sequence^d^	1

a Sub-vector of 36 variables standing for the size of internal loops in the candidate mature sequence region in cases that unpaired nucleotides on the 5′ arm varies within 1–6 nt and unpaired nucleotides on the 3′ arm varies within 1–6 nt;

b Sub-vector of 4 variables, including the number of bulges and the number of unpaired nucleotides involved in the bulges on both the 5′ and 3′ arms;

c The position of the first paired nucleotides from the non-loop side of the secondary structure of pre-microRNA;

d The number of nucleotides between the 5′ end and the candidate Dicer processing site.

### Performance of MiRmat on the Prediction of Mature microRNA

We compared the accuracy of MiRmat for mature microRNA prediction with three existing methods, proMIR, MatureBayes and MaturePred [43]. Performance of these tools is shown in Table 3, 4 and 5.

**Table 3:** Performance of tools on the prediction of mature microRNA sequence.

	5′-strand^a^				3′-strand^b^			
	Drosha site		Dicer site		Drosha site		Dicer site	
	mean	SD^c^	mean	SD^c^	mean	SD^c^	mean	SD^c^
*MiRmat_v4*	**1.64**	**1.82**	**2.25**	**2.24**	**1.47**	**1.98**	2.00	**1.80**
*proMIR_v4*	1.96	2.56	2.47	3.26	2.13	2.70	**1.60**	2.14

a 5′-strand indicates that the mature sequence locates on the 5′ arm of the stem-loop structure of microRNA;

b 3′-strand indicates that the mature microRNA sequence locates on the 3′ arm.

c Standard deviation based on the entire population (SDP) was used for the 5-fold cross-validations.

**Table 4:** Performance comparison between MatureBayes and MiRmat.

Distance from Truth^a^	0	±1	±2	±3
*MatureBayes* (%)	12.75	34.20	45.51	59.13
*MiRmat* (%)	**20.35**	**39.29**	**54.39**	**60.35**

a the distance between predicted start position of mature microRNA and that of actual mature microRNA.

**Table 5:** Performance comparison between MaturePred and MiRmat.

Distance from Truth^a^	0	±1	±2	±3
*MaturePred*(%)	21.21	36.36	48.48	57.57
*MiRmat* (%)	**23.03**	**45.45**	**59.39**	**69.70**

a the distance between predicted start position of mature microRNA and that of actual mature microRNA.

#### Comparison with proMIR

To compare the performance of MiRmat with proMIR [9], we applied the two tools to 136 human microRNAs (miRBase version 4.0) through a 5-fold cross-validation as did in the previous study [9]. Two parameters were introduced to measure the performance: the mean of the absolute distance between the predicted and true sites and the standard deviation. Results are shown in Table 3. Here the standard deviation based on the entire population (SDP) was used for the convenience of comparison with the previous data provided by proMIR. The bold numbers highlight the improved performances in terms of each parameter.

In general, MiRmat showed higher accuracy than proMIR for both Drosha and Dicer processing site predictions. The performance on Dicer sites is not as favorable as that compared to the Drosha sites. This may be due to the accumulation of errors at the two ordinal prediction stages.

#### Comparison with MatureBayes

To compare MiRmat with MatureBayes, another mature microRNA identification tool developed recently, we included human and mouse microRNAs in miRBase 10.1 as the training dataset and those in miRBase 11–14 as the test set, which is the same as that for MatureBayes [31]. Results are shown in Table 4. As did in MatureBayes, we measured the performance of both tools under different deviations which is named ‘distance from truth’ in Table 4. In MatureBayes, it means the prediction error at 5′ end of mature microRNAs. In MiRmat, ‘distance from truth’ represents the error of Drosha processing site for microRNAs on 5′ arm. While for the microRNAs on 3′ arm, it means the error of Dicer processing site. The mean distance and standard deviation from MiRmat are 3.607 nt and 5.007 nt respectively, which are lower than 5.298 nt and 7.817 nt resulted from MatureBayes. A p-value of 1.283×10^−4^ was obtained by paired *t*-test which indicates that the observed difference between the two methods is significant.

The distance distribution within 3 nt was presented in Table 4 in which MiRmat shows higher detection rate than MatureBayes in the prediction of the start point of mature microRNA sequence within this error range. As there are 49 microRNAs removed from miRBase according to miRBase 17 released this year, the test set we actually used is 272 precursors (285 mature microRNAs). Out of them, 58 mature microRNA sequences were predicted precisely at the start position.

#### Comparison with MaturePred

We further compare MiRmat with a more recent mature microRNA identification tool, MaturePred. The newly found human and mouse microRNAs in miRBase 18 were retrieved as the test set. Results are shown in Table 5. In order to make a consistent evaluation, the output results from MaturePred with the highest probability at each arm were taken into assessment. This is different from the way that the author of MaturePred took to evaluate the performance of their own tool [43], in which the top 10 mature microRNA candidates with highest probabilities for each precursor microRNA were taken. The minimum distance between each candidate and the actual mature microRNA was measured as the prediction deviation.

Like the other assessment, the performance of both tools were measured by the parameter ‘distance from truth’ in Table 5. This parameter represents the error of Drosha processing site for microRNAs on 5′ arm, for the microRNAs on 3′ arm, it means the error of Dicer processing site. The mean distance and standard deviation from MiRmat are 2.901 nt and 3.980 nt respectively, which are better than 4.963 nt and 6.328 nt resulted from MaturePred. The significance of the comparison is measured by p  = 7.757×10^−6^. As presented in Table 5, the rate of identifying mature microRNA position approaches 70% by MiRmat which is over ten percent higher than the performance of MaturePred when an error of 3 nt is allowed.

## Discussion

In this study, we developed the tool, MiRmat, for the identification of mature microRNA sequence from microRNA hairpins via sequentially predicting the Drosha and Dicer processing sites. Based on the evaluation of MiRmat, we have demonstrated that MiRmat has improved the performance in both the accuracy and correctness rate of mature microRNA prediction. In addition, MiRmat is applicable to more organisms other than human and mouse, for them most of the recent methods were designed. The results from evaluations indicate that the free energy profile involved in this method is an efficient feature for the recognition of mature microRNA and is general to a wide range of organisms among vertebrates.

MiRmat employed the ‘free energy profile’ as the feature to predict Drosha processing site and gained high accuracy, which indicates that the energy distribution pattern downstream Drosha processing site may serve as the key determinant for Drosha to recognize its cutting point. This is consistent with the previous conclusion [36]. This type of energy pattern is actually a combination of RNA structure and sequence features because the energy of a ‘stack face’ is determined by the structure of this face and the nucleotides that are involved. Moreover, our analysis on the whole set of microRNAs from 12 species showed that this energy distribution pattern could be conserved among vertebrates, while the invertebrates presented a significantly different pattern. This discovery simply extended the application of MiRmat to a variety of vertebrate species.

MiRmat uses ‘structural features’ for predicting Dicer processing site. Based on our finding, although the length of microRNA mature sequence serves as the key determinant for the Dicer processing site, the structural features alter the precise position of the Dicer site. In general, the recognition and interaction between the enzyme and substrate are determined by the three dimensional structures of both molecules. However, the mechanism that determines the Dicer processing site on pre-microRNA hairpin is very complicated [44]. Limited by the existing technologies, we only used secondary structure to develop the prediction method in this study.

MiRmat employs the principle of molecular interaction to predict mature microRNA sequences. The method is based on the similarity of free energy and structural features among vertebrate microRNA hairpins rather than on microRNA sequence conservation. This ensures the applicability of MiRmat to the characterization of microRNAs in a variety of vertebrate genomes including the newly sequenced species. MiRmat was designed according to the biogenesis of mature microRNA and each of the features used in this method represents a definite biological meaning. So the significance of features and parameters from each step of the prediction can provide new clues for deducing the mechanism of mature microRNA biogenesis. For example, base-pairing in pre-microRNAs may differ from that in pri-microRNAs after Drosha processing and being transported from the nucleus to the cytoplasm in the form of a single chain. However, this kind of structural change can hardly be directly reflected by the pri-microRNA sequence. Actually, ignoring this change may limit the accuracy of mature microRNA sequence identification. Meanwhile, MiRmat has been constructed to include three functional parts, the Drosha processing site prediction, the prediction of Dicer site on pre-microRNA sequences, and microRNA mature sequence prediction. These functional modules provide a useful selection for the task of microRNA annotation and satisfy the design requirements from a wide variation of experimental studies.

Compared with Microprocessor SVM which involves more than 600 features for Drosha site prediction, MiRmat only uses 21 features of thermodynamics and length of sequence while achieving the same performance. The successful prediction of Drosha site by using a limited number of features from free energy profile not only demonstrates the free energy profile derived in this study as an effective determinant of Drosha interaction to pri-microRNA, but also implies a general enzyme-RNA interaction mechanism among microRNAs of human as well as other vertebrate species.

In the prediction of mature microRNA sequence, MiRmat outperforms the other current methods, proMIR and MatureBayes, in that it showed much better prediction accuracy and correctness rate. Note that the sequence region spanning 10–20 nt downstream pre-microRNA plays an important role in determining Drosha processing site, we suspect that MatureBayes may missed some information by only including the flanking region of 9 nucleotides of mature microRNA.

In summary, MiRmat reported in this paper presents several advantages in predicting mature microRNA sequence. First, it is based on the common pattern of free energy profile and thus is adaptable to a wide range of vertebrate microRNA analysis; second, it achieved a better accuracy and rate of correctness in mature microRNA prediction; finally, it is designed according to the biogenesis of mature microRNA and provides a choice of access at each of the maturation steps including the prediction of Drosha processing site, the prediction of Dicer site and the prediction of the whole mature microRNA sequence, which will benefit data analysis for various experiments. For example, MiRmat may help to filter the sequencing data produced from Solexa/Illumina and find out the most probable microRNAs from the reads by making mature microRNA sequence prediction based on the sequencing data and the genome regions they are embedded.

Further studies could be focused on the identification of mature microRNA sequences located in irregular structures, for example, which are located at the terminal loop of microRNA hairpins. In addition, limited by present techniques, only secondary structure was considered in the development of MiRmat. We believe that although the secondary structure reflects the three-dimensional structure of RNA to a large extent, direct involvement of the spacial data will hopefully increase the prediction ability for Dicer site determination.

## Materials and Methods

### Predicting Drosha Processing Sites

**Figure 4:**
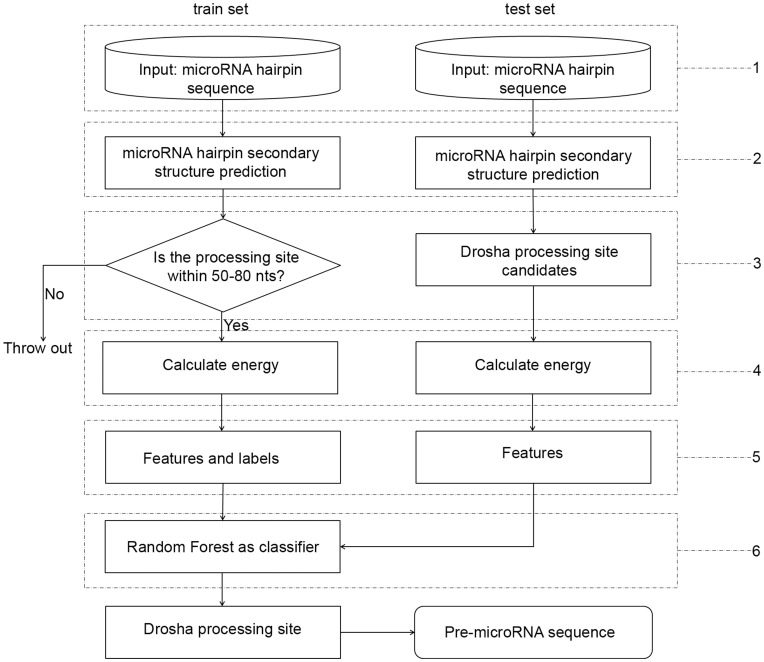
Flow chart for the method of Drosha processing site prediction.

The flow chart of Drosha processing site prediction in MiRmat is illustrated in Fig. 4. First, the free energy profiles of known microRNA hairpins were calculated. Second, the features and labels were extracted from the sequences and the energy profiles. Then, these features and labels were used for training the Random Forest classifier. Finally, the Random Forest classifier was applied for predicting the Drosha processing site of new microRNA hairpins with extracted features.

#### Data retrieval

MicroRNA sequences and genomic positions were downloaded from miRBase. The microRNA hairpin sequences were retrieved with a window of 130 nt from Ensembl genome database (Ensembl release 53 of March 2009 [45]), centering on the stem-loop structure provided by miRBase.

The training set of 1944 vertebrate microRNAs was from miRBase [46] version 9.2, in which 119 repeated samples have been excluded. The new vertebrate microRNAs appeared in version 12.0 were used as the test set, which included 1204 microRNAs. To insure the independency between the training set and the testset, hairpin sequences with a similarity of over 90% to those in the train set have been excluded.

#### RNA secondary structure prediction

RNAfold of Vienna package 1.8 [41] with default parameters and thermodynamic data from Turner group [47], [48], [49] were applied in the prediction of RNA secondary structures.

#### Definition of the candidate Drosha processing site

As previously outlined by Helvik, S.A. et al. (2007) in Microprocessor SVM, all of the Drosha processing sites were defined on the 5′ arm of microRNA stem-loop structures with the processing site of the 3′ arm overhanging by 2 nt. If the mature sequence locates on the 5′ arm, then the Drosha processing site was defined by the 5′ end of the mature microRNA sequence. If there are mature sequences on both arms, then the Drosha site was still defined by the 5′ end of the mature microRNA sequence located on 5′ arm. In cases of microRNAs with mature sequences only on 3′ arms, the Drosha processing sites were still defined on the 5′ arms using the location that results in a 2 nt-overhang at the 3′ end of the mature microRNA sequence.

According to our statistics on miRBase 9.2, the lengths of about 96% of the pre-microRNAs are within 50–80 nt. Therefore, the candidate pre-microRNAs were defined to be within this range, as adapted by the previous study [13].

#### Calculation of free energy profiles

**Figure 5:**
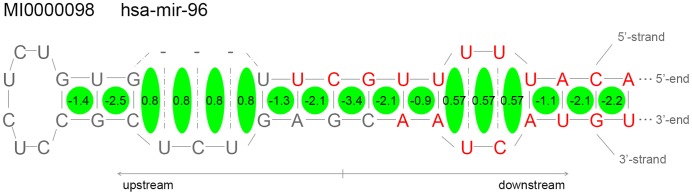
Illustration of the method for calculating the free energy profile of the microRNA stem-loop structures. Part of the secondary structure of microRNA MI0000098 hairpin is shown. Each green ellipse represents one single ‘stack face’. The value in each of the green ellipses indicates the free energy of this face. The nucleotides in red belong to the mature sequence of this microRNA.

Fig. 5 illustrates the method for calculating the free energy profiles of the stem-loop structure derived from pri-microRNAs. Taking MI0000098 as an example, the folded microRNA was divided into individual neighbors [49] according to the stacking status. The bulges and internal loops were further split into pieces for each of the nucleotides whether paired or not. We called these pieces the ‘stack faces’. Each single ‘stack face’ corresponds to a free energy that represents the thermodynamic stability of this face. The thermodynamic parameters of the stack faces were from Turner group [49], and those faces split from bulges and loops were assigned the average values of the total energy of the corresponding stacks.

#### Feature vector for predicting Drosha processing sites

According to the result of a 10-fold cross validation on the free energy profiles (data not shown), we chose the energy values of 20 stack faces (including the one corresponding to the candidate Drosha processing site itself) downstream the candidate Drosha processing site to construct the feature vector. In addition, the length of the candidate pre-microRNA was also included in the vector. Therefore, every candidate processing site was represented by a feature vector of 21 variables.

#### Classifier

Random Forest (RF) [50] was used as the predictive model to pick out the true Drosha/Dicer processing site from the candidate processing sites. RF is a well-known ensemble method [51] in the machine learning community which combines a series of decision trees and shows good generalization performance. RF runs under three main steps. First, new training sets are drawn from the original training set by sampling with replacement. Then, for each new training set, a non-pruned decision tree is growing using a random feature selection method to inject randomness to classic decision tree models [50]. Finally, all the decision tree models are applied to the test sample and outputs are produced. The final prediction results can be then computed from these results by some ensemble schemes, e.g., the most popular majority voting method. With respect to our task, each sample is the feature vector charactering a candidate processing site produced either by Drosha or Dicer. In RF, each decision tree outputs a decision on whether the test sample is the true processing site. Thus, the number of the trees that vote for the sample to be the true processing site is adopted as the prediction score in this paper. For a microRNA sequence, the position with the highest prediction score is taken as the predicted processing site.

In this work, the RF algorithm is implemented by weka [52] (version 3.6.1).

### Predicting Dicer Processing Sites

The Drosha processing site only defines one end of the mature microRNA sequence. In order to find the other end of the mature microRNA, we searched upstream of the Drosha site to identify the Dicer processing site. The procedures of applying Random Forest classifier for predicting the Dicer site were similar to those procedures for predicting Drosha processing site (Figure S3).

#### Definition of the candidate Dicer processing site

Similar to our assumptions for the Drosha candidate processing sites (refer to Section 2.1.3), all of the Dicer processing sites were also defined on the 5′ arm of the pre-microRNAs with a 2 nt-overhang to the processing site on the 3′ arm. If a microRNA hairpin carries the mature sequence on the 5′ arm or on both arms, the Dicer processing site was defined as the 3′ end of the mature sequence of the microRNA. If the microRNA only had its mature sequence on the 3′ arm, the Dicer processing site on the 5′ arm was located at a position with a 2 nt overhanging relative to the 5′ end of the mature sequence.

According to previous statistics, nearly all mature microRNAs (99.9%) in the training set are within 16–25 nt. Therefore, the candidate Dicer processing sites were defined to be within this range.

#### Feature vector for predicting Dicer processing site

A total of 42 features were derived from a statistical analysis on the relation of pre-microRNA hairpin structures to the Dicer processing site in the training set. The duplex between the end of the stem of the pre-microRNA hairpin and the candidate Dicer processing site was termed a ‘candidate mature sequence region’. The features belong to 4 categories as been listed in Table 3. The size of internal loops were restricted to 6 nt based on the statistical results from the training set.

## Supporting Information

Figure S1 **Energy distribution patterns of the microRNA stem-loops of 12 species.** The free energy distributions along the stem of the microRNA hairpin structure of 12 organisms were plotted, including 10 vertebrates and 2 invertebrates. The horizontal axis represents the distance of a stack face away from the Drosha cutting site. Zero indicates the stack face at the Drosha cutting site. Minus refers to upstream (i.e., the direction to the loop of hairpin). It is clear that all vertebrates have, to some extent, similar free energy distribution pattern (e.g., an low free energy distribution in the region of 10–15 nt and an energy peak in the region of 15–20 nt), with fluctuations though. From the positions 0 to 19, the correlation coefficients of the energy distribution between H. sapiens(hsa) and the other vertebrates, *B. taurus(bta)*, *D. rerio(dre)*, *M. domestica(mdo)*, *M. mulata(mml)*, *M. musculus(mmu)*, *P. troglodytes(ptr)*, *R. norvegicus(rno)*, *G. gallus(gga)*, *X. tropicalis(xtr)* are 0.8977, 0.9227, 0.9528, 0.9086, 0.9905, 0.9367, 0.9741, 0.9207, 0.9516 respectively. While the invertebrates, *C. elegans(cel)* and *D. melanogaster(dme)*, do not have this characteristic pattern and the distributions of these two invertebrates are quite different from those of vertebrates.
(DOC)

Figure S2 **Rate of correct predictions of Drosha processing site in a more stringent evaluation.** ‘Deviation (nt)’ means the distance between the predicted Drosha processing site and the true site. (A) Performance on microRNAs of 10 vertebrates in the test dataset (*M. domestica, H. sapiens, M. mulata, M. musculus, P. troglodytes, R. norvegicus, G. gallus, X. tropicalis, O. anatinus, C. familiaris*); (B) Performance on *O. anatinus* microRNA data set.
(DOC)

Figure S3 **Flow chart for developing the method of Dicer processing site prediction.**
(DOC)
