# Supplementary figures and images for: MiRmat: Mature microRNA Sequence Prediction
Source: PLoS One. 2012 Dec 27;7(12):e51673. doi: 10.1371/journal.pone.0051673 (PMC3531441; doi:10.1371/journal.pone.0051673)

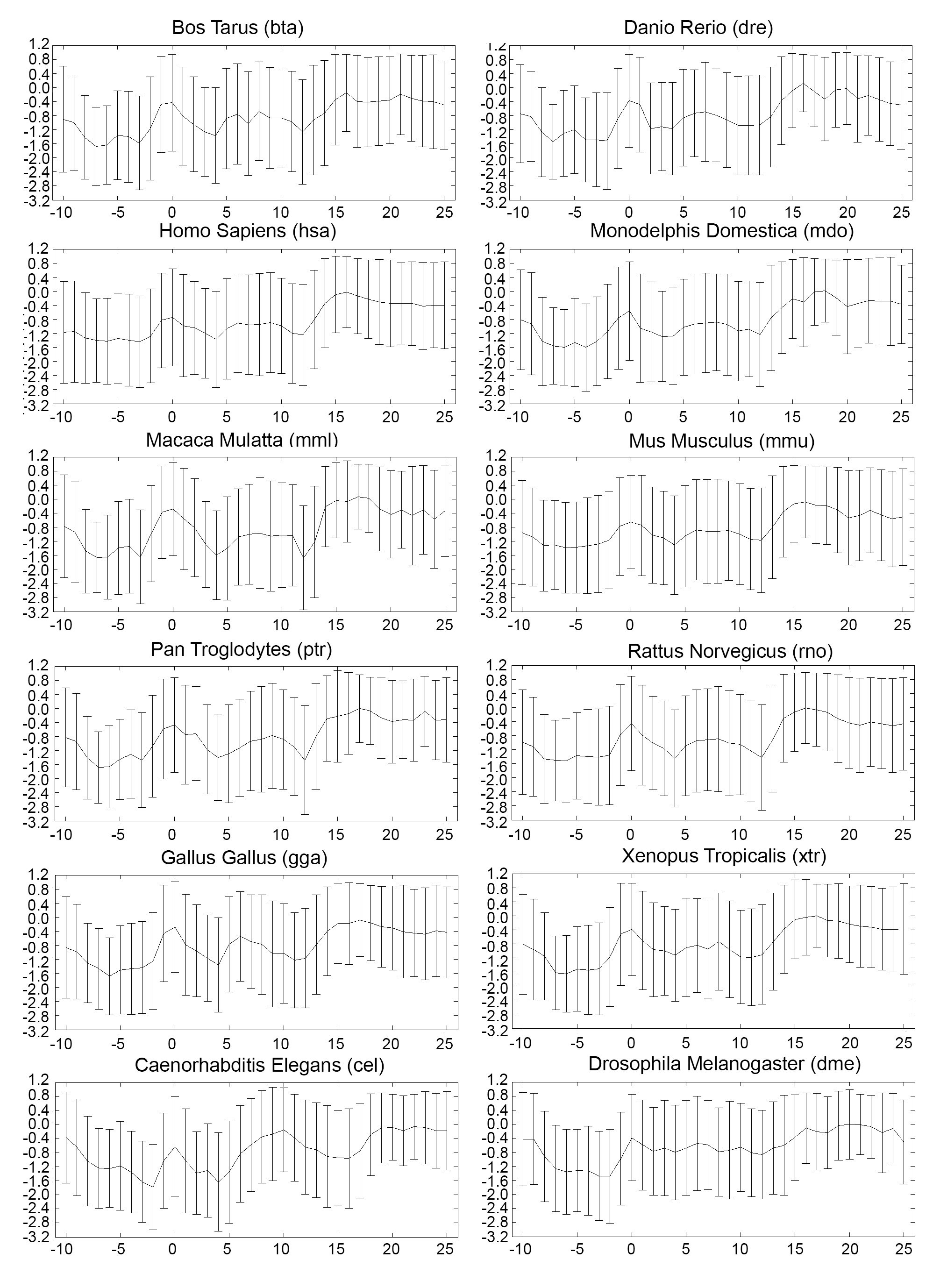

Supplement: Figure S1 — Energy distribution patterns of the microRNA stem-loops of 12 species. The free energy distributions along the stem of the microRNA hairpin structure of 12 organisms were plotted, including 10 vertebrates and 2 invertebrates. The horizontal axis represents the distance of a stack face away from the Drosha cutting site. Zero indicates the stack face at the Drosha cutting site. Minus refers to upstream (i.e., the direction to the loop of hairpin). It is clear that all vertebrates have, to some extent, similar free energy distribution pattern (e.g., an low free energy distribution in the region of 10–15 nt and an energy peak in the region of 15–20 nt), with fluctuations though. From the positions 0 to 19, the correlation coefficients of the energy distribution between H. sapiens(hsa) and the other vertebrates, B. taurus(bta), D. rerio(dre), M. domestica(mdo), M. mulata(mml), M. musculus(mmu), P. troglodytes(ptr), R. norvegicus(rno), G. gallus(gga), X. tropicalis(xtr) are 0.8977, 0.9227, 0.9528, 0.9086, 0.9905, 0.9367, 0.9741, 0.9207, 0.9516 respectively. While the invertebrates, C. elegans(cel) and D. melanogaster(dme), do not have this characteristic pattern and the distributions of these two invertebrates are quite different from those of vertebrates. (DOC) [file pone.0051673.s001.doc]

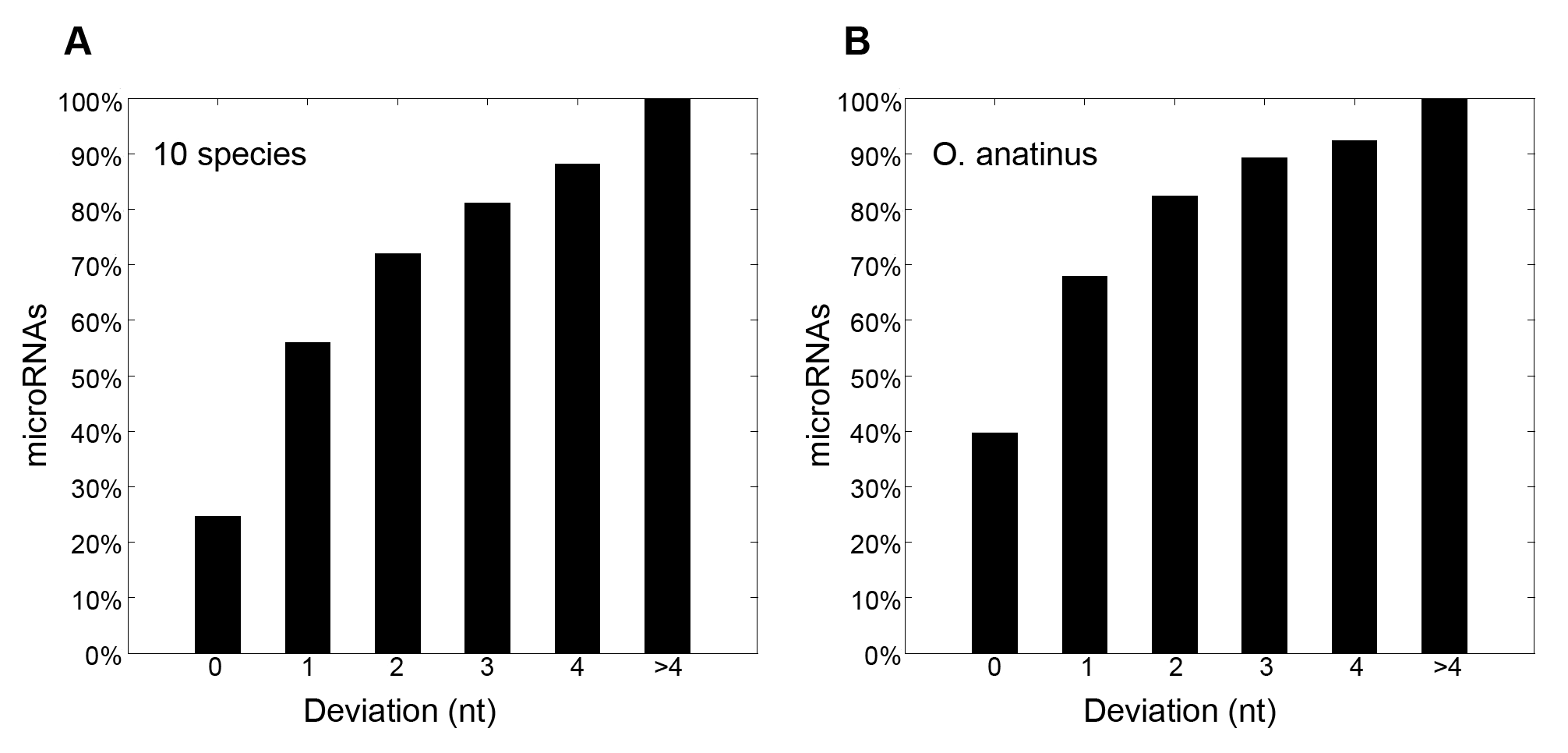

Supplement: Figure S2 — Rate of correct predictions of Drosha processing site in a more stringent evaluation. ‘Deviation (nt)’ means the distance between the predicted Drosha processing site and the true site. (A) Performance on microRNAs of 10 vertebrates in the test dataset (M. domestica, H. sapiens, M. mulata, M. musculus, P. troglodytes, R. norvegicus, G. gallus, X. tropicalis, O. anatinus, C. familiaris); (B) Performance on O. anatinus microRNA data set. (DOC) [file pone.0051673.s002.doc]

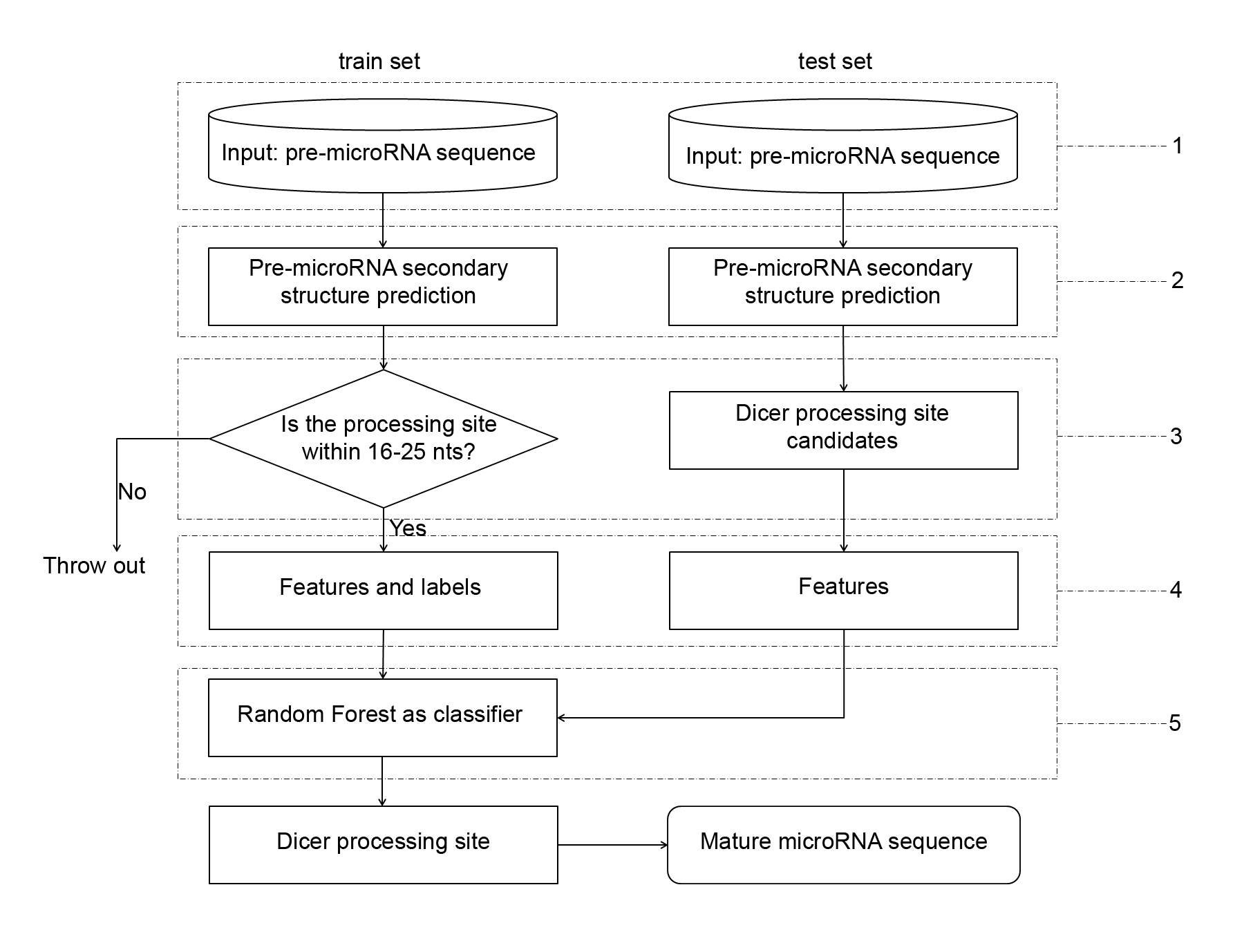

Supplement: Figure S3 — Flow chart for developing the method of Dicer processing site prediction. (DOC) [file pone.0051673.s003.doc]
